# A comment to ‘Efficacy of surgical intervention over conservative management in intertrochanteric fractures among nonagenarians and centenarians: a prospective cohort study’

**DOI:** 10.1097/JS9.0000000000001740

**Published:** 2024-05-29

**Authors:** Jiaan Lu, Guanhu Yang, Hao Chi, Qinglai Wang

**Affiliations:** a School of Clinical Medicine, The Affiliated Hospital, Southwest Medical University, Luzhou, Sichuan; b Orthopedics and Traumatology Department of TCM, Wenzhou TCM Hospital of Zhejiang Chinese Medical University, Wenzhou, People’s Republic of China; c Department of Specialty Medicine, Ohio University, Athens, Ohio, USA

*Dear Editor,*

I am writing this letter to express my gratitude to Guo *et al*.^1^ for their significant research titled ‘Efficacy of surgical intervention over conservative management in intertrochanteric fractures among nonagenarians and centenarians: a prospective cohort study.’ Given the global aging population, this research addresses a critical and increasingly relevant issue: the optimal treatment strategy for hip fractures in individuals aged 90 and above.

The authors’ prospective cohort design and comprehensive data collected from the CPMHF database provide a solid foundation for their findings. Their detailed and thorough assessment of comorbidities using the modified Elixhauser Comorbidity Measure (mECM), along with the application of advanced statistical methods such as restricted cubic spline analysis and propensity score matching, enhance the reliability and depth of the study. The inclusion of long-term follow-up data up to 6 years is particularly commendable and offers valuable insights into the outcomes for this vulnerable population.

The results of this study emphasize the substantial benefits of surgical intervention for non-elderly individuals and centenarians with hip fractures. Even when considering the competing risk of death, surgical treatment is associated with significantly better outcomes in terms of survival rates and a lower incidence of severe complications. Notably, patients with high mECM scores (HMS ≥3) showed significant benefits from surgical intervention, highlighting the importance of individualized treatment plans based on comprehensive comorbidity assessments.

The authors also identified subgroups, including females, uninsured patients, and those with spouses, who might gain greater advantages from surgical intervention. These insights are crucial for tailoring treatment strategies to improve the prognosis of specific patient groups.

Although the advantages of this study are evident, some limitations are also worth noting.

The single-center design may limit the generalizability of the findings, and excluding femoral neck fractures may restrict the applicability of the results to all types of hip fractures. It also cannot verify the best treatment options for different types of fractures. Future studies should consider multicenter research with larger sample sizes to validate these findings across different healthcare settings and populations. Multicenter studies allow for replication of findings, which is crucial for validating results and ensuring they are not due to chance or specific conditions of a single site^2^.

Additionally, the study could benefit from exploring the impact of other factors, such as muscle strength, osteoporosis, and medication use, on the outcomes. Including these variables could provide a more comprehensive understanding of the factors affecting rehabilitation and survival in this age group^3^.

There might also be a selection bias in the study, as the research sample primarily comes from a tertiary referral institution of a university-affiliated hospital, where patients might have more complex conditions or severe fractures. Future research should consider a broader case selection, including patients from primary and community hospitals, to reduce selection bias and discover more findings^4^.

For very elderly patients, balancing treatment costs and benefits is a crucial consideration, that is, from a cost-effectiveness perspective. The article also considers the impact of family factors on the prognosis of elderly patients and provides an analysis of the reasons. Future research should include economic analyses to evaluate the feasibility and cost-effectiveness of surgical versus conservative treatment^5^.

The study emphasizes the importance of a comprehensive management model but does not discuss the impact of specific management measures in detail. Future research could analyze in more depth which specific management measures (such as preoperative optimization, postoperative rehabilitation, etc.) have a significant impact on the outcomes to improve clinical practice.

In summary, the study by Guo *et al*.^1^ has made a valuable contribution to the literature on the treatment of hip fractures in very elderly patients. Their findings advocate for the benefits of surgical intervention and emphasize the importance of individualized treatment plans based on comorbidity assessments. Addressing the identified limitations and expanding the scope of future research can further enhance the applicability and impact of these results.

Finally, I sincerely thank the authors for their outstanding and significant work in advancing our understanding of the optimal treatment strategies for hip fractures in non-elderly individuals and centenarians. We highly commend and recognize the value and significance of their research findings.

## Ethical approval

Not applicable.

## Consent

Not applicable.

## Source of funding

This project was supported by the Research on Chinese Medicine Nutrition and Rehabilitation Intervention Strategies for Osteoporosis Patients in Wenzhou Community (No. 2018ZY017).

## Author contribution

J.L., G.Y., H.C., and Q.W.: conceptualization, methodology, validation, formal analysis, investigation, resources, data curation, writing – original draft, writing – review and editing, supervision, and project administration.

## Conflicts of interest disclosure

There are no conflicts of interest.

## Research registration unique identifying number (UIN)

Not applicable.

## Guarantor

Jiaan Lu, Guanhu Yang, Hao Chi, and Qinglai Wang.

## Data availability statement

Not applicable.

## Provenance and peer review

Not applicable.

## Assistance with the study

Not applicable.
